# Validation of a simplified scatter correction method for 3D brain PET with ^15^O

**DOI:** 10.1007/s12149-016-1114-7

**Published:** 2016-08-17

**Authors:** Masanobu Ibaraki, Keisuke Matsubara, Kaoru Sato, Tetsuro Mizuta, Toshibumi Kinoshita

**Affiliations:** 1 Department of Radiology and Nuclear Medicine, Akita Research Institute of Brain and Blood Vessels, 6-10 Senshu-Kubota Machi, Akita, 010-0874 Japan; 2 Medical System Division, Shimadzu Corporation, Kyoto, Japan

**Keywords:** PET, Scatter correction, Brain, ^15^O

## Abstract

**Objective:**

Positron emission tomography (PET) enables quantitative measurements of various biological functions. Accuracy in data acquisition and processing schemes is a prerequisite for this. The correction of scatter is especially important when a 3D PET scanner is used. The aim of this study was to validate the use of a simplified calculation-based scatter correction method for ^15^O studies in the brain.

**Methods:**

We applied two scatter correction methods to the same ^15^O PET data acquired from patients with cerebrovascular disease (*n* = 10): a hybrid dual-energy-window scatter correction (reference method), and a deconvolution scatter correction (simplified method). The PET study included three sequential scans for ^15^O-CO, ^15^O-O_2_, and ^15^O-H_2_O, from which the following quantitative parameters were calculated, cerebral blood flow, cerebral blood volume, cerebral metabolic rate of oxygen, and oxygen extraction fraction.

**Results:**

Both scatter correction methods provided similar reconstruction images with almost identical image noise, although there were slightly greater differences in white-matter regions compared with gray matter regions. These differences were also greater for ^15^O-CO than for ^15^O-H_2_O and ^15^O-O_2_. Region of interest analysis of the quantitative parameters demonstrated that the differences were less than 10 % (except for cerebral blood volume in white-matter regions), and the agreement between the methods was excellent, with intraclass correlation coefficients above 0.95 for all the parameters.

**Conclusions:**

The deconvolution scatter correction despite its simplified implementation provided similar results to the hybrid dual-energy-window scatter correction. We consider it suitable for application in a clinical ^15^O brain study using a 3D PET scanner.

## Introduction

Positron emission tomography (PET) enables the quantification of various biological functions, and the use of ^15^O-labeled compounds allows estimates of cerebral blood flow (CBF) and oxygen metabolism [1–3]. The validity of quantitative results depends on the accuracy of the PET acquisition and data processing schemes, which include corrections for detector efficiency, scanner dead-time, random coincidences, photon attenuation, and photon scatter. Currently, all the commercially-available PET scanners employ 3D-acquisition mode in which scatter coincidences are greater than in conventional 2D-acquisition mode, where the scatter effect is marginal. A scatter correction is, therefore, essential to achieve accurate PET data with 3D scanners [4]. However, it is generally difficult to validate the accuracy of the scatter correction for human PET scans, because scatter-free ground-truth results are not known.

PET scatter correction methods fall into two categories: energy-window-based methods [5, 6] and calculation-based methods [4, 7–10]. We have previously validated a 3D-dedicated scanner with a hybrid dual-energy-window (HDE) scatter correction (one of the energy-window-based methods), by performing a head-to-head comparison with a conventional 2D scanner on a ^15^O study in the brain [6, 11]. However, as the energy-window-based method requires a special setting for two (or more) energy windows and has strict stability requirements because of the narrower energy window [6], calculation-based methods, including a single scatter simulation (SSS) method [9, 10], are utilized in most major clinical PET scanner products. A common drawback of the calculation-based methods is an inability to correct for scatter events originating from radioactivity outside of the field of view (FOV). This may cause a substantial effect in ^15^O PET of the brain, where strong radioactivity exists outside of the brain, such as in the lungs, heart, and airways [12, 13].

The aim of this study was to validate the use of the calculation-based method for a clinical PET study using ^15^O. We applied a convolution-subtraction scatter correction [4, 7], and compared it with the HDE scatter correction, which we considered as the reference method [11]. The convolution-subtraction method estimates the scatter distribution by convolving the measured sinograms with the scatter kernel. The estimated scatter is subsequently subtracted to obtain the corrected, scatter-free sinograms. This process can be seen as an inverse of the convolution, that is it can be considered a deconvolution; therefore, we refer to the method as deconvolution scatter correction (DEC) [7] in this paper.

## Materials and methods

### Subjects

PET data from 10 sequential cases acquired between April and June 2014, were analyzed retrospectively. All the patients had occlusion or stenosis of the internal carotid artery (ICA; *n* = 5) or middle cerebral artery (*n* = 5) as demonstrated by MR or CT angiography. This retrospective study was approved by the local ethics committee (No. 15-11, Ethics Committee of Research Institute for Brain and Blood Vessels-Akita).

### PET scanner

A SET-3000GCT/M (Eminence SOPHIA; Shimadzu Corp., Kyoto, Japan) dedicated to the 3D-acquisition mode was used [11, 14]. The scanner consisted of 30 gadolinium oxyorthosilicate crystal rings, providing 59 slices, each with a thickness of 2.6 mm. The axial FOV was 156 mm. The lower limit of the energy window (a determinant of the amount of scatter) was set to 400 keV. The scanner was operated in a 64-bit list mode. Three-dimensional sinograms were converted to 2D sinograms using a Fourier rebinning algorithm (FORE). Scatter correction was performed on the 2D sinograms following the description below. Attenuation correction was applied to the scatter-corrected 2D sinograms via transmission scanning (3 min) using a ^137^Cs point source and a bismuth germanate transmission detector ring, coaxially attached to the emission detector rings. Reconstruction by filtered back projection with a 6 mm FWHM 3D Gaussian filter resulted in an effective in-plane resolution of 7 mm. All reconstructed images consisted of 59 slices of 128 × 128 voxels, with a voxel size of 2.0 × 2.0 × 2.6 mm.

### PET protocol

Three emission scans were performed sequentially with inhalation of ^15^O-CO, inhalation of ^15^O-O_2_, and injection of ^15^O-H_2_O, with 15-min intervals between the scans [15, 16]. The patient’s head was fixed using pads and a Velcro band tightened around the head and head holder [17]. A removable neck-shield consisting of 7 mm thick lead plates (corresponding to 70 % attenuation of 511 keV gamma rays) was used to reduce random and scatter coincidences attributable to radioactivity outside of the FOV [13].

Calibration between the PET scanner and a well counter was performed by scanning a cylindrical phantom (15 cm in diameter) filled with a ^68^Ga aqueous solution, and subsequently, a beta detector for measuring an arterial input function was calibrated to the well counter. In this procedure, reconstruction images were generated with HDE and DEC, and calibration factors were separately calculated for HDE and DEC. Scanner count rates with ^22^Na point source were measured every day to check the stability of the scanner and to assure the accuracy of the HDE scatter correction, and we confirmed that for the examinations analyzed in the study (*n* = 10), the UEW count rates were within 5 % from the baseline (the most recent scanner calibration).

The ^15^O-H_2_O PET study to measure CBF used a 3 min scanning duration with a simultaneously initiated 2 min intravenous infusion of ^15^O-H_2_O (0.37 GBq) by an automatic injector device [3, 18]. The arterial input function was determined with the beta detector system [19], and the CBF was calculated using the autoradiographic method [3, 18]. The ^15^O-CO PET study to measure cerebral blood volume (CBV) used a 4 min scan initiated 3 min after a 1 min inhalation of ^15^O-CO gas (2.13 ± 0.20 GBq) [20]. The ^15^O-O_2_ PET study with a 3 min scan initiated simultaneously with 1.5 min inhalation of ^15^O-O_2_ (3.39 ± 0.51 GBq) was performed to measure oxygen extraction fraction (OEF) and cerebral metabolic rate of oxygen (CMRO_2_) [2]. The arterial input function was determined in the same way as the ^15^O-H_2_O PET scan.

### Scatter correction

The acquired 3D sinograms were sorted into 2D sinograms using FORE. The subsequent 2D sinograms were then scatter-corrected using both the HDE and DEC routines. HDE scatter correction based on a dual-energy-window acquisition was used as a reference for the comparison. The details of the method have been described previously [6, 11]. In brief, we estimated scatter components in the standard energy window (SEW; 400–624 keV) by combining SEW data and upper energy-window (UEW) data (512–624 keV), in which the scatter contribution is relatively small, and hence, correctable by the DEC method.

**Fig. 1:**
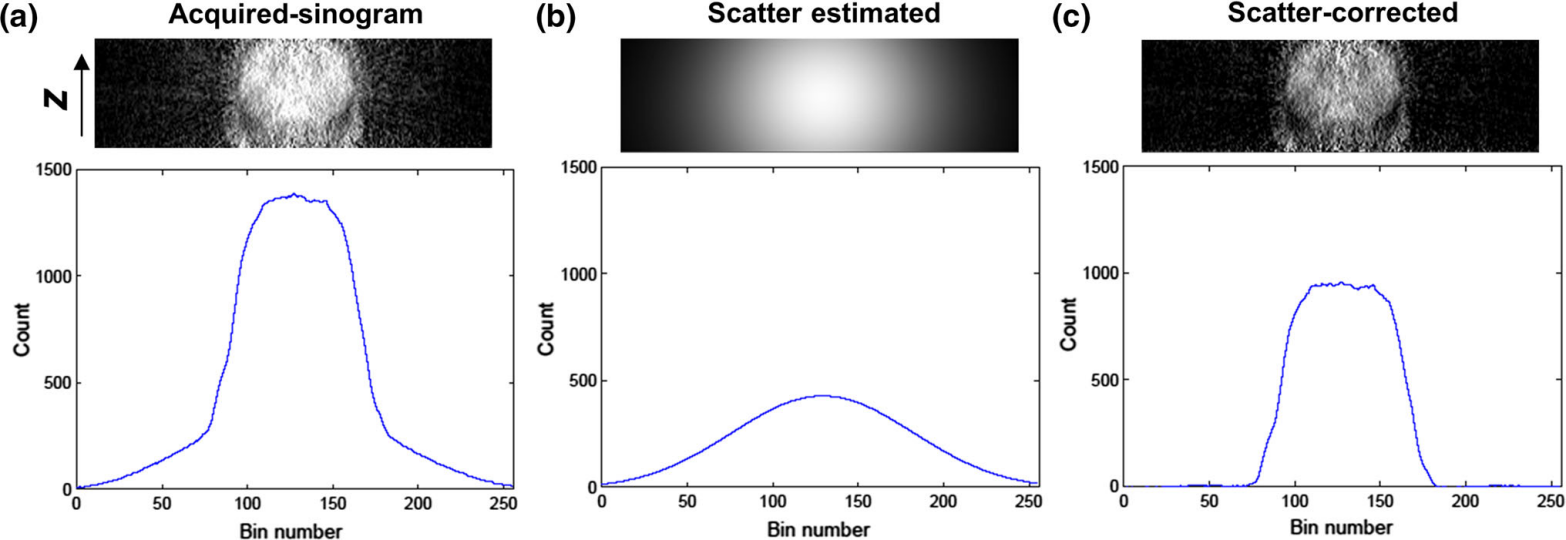
Schematic view of deconvolution scatter correction: scatter components in SEW (**b**) is estimated by convolving the SEW sinograms (**a**) with the scatter kernel, is subsequently subtracted to obtain the scatter-corrected SEW sinograms (**c**). *SEW* standard energy window

The convolution-subtraction scheme was applied in the DEC method [4, 7]: we estimated the scatter components in SEW by convolving the SEW sinograms with the scatter kernel, and subsequently subtracted the estimated scatter to obtain the scatter-corrected SEW sinograms (Fig. 1). The scatter kernel (*S*) is defined as a low-pass filter in the spatial frequency domain of the 2D projection plane of the sinograms (radial-direction × z-direction for each view angle):

$$S(\overrightarrow {f} ) = 1/(1 + \alpha \exp (\beta \left| {\overrightarrow {f} } \right|^{2} )) ,$$

where *f* represents spatial frequency and (*α*, *β*) is a parameter set that determines the amplitude and shape of the scatter kernel. This function was empirically selected to represent the scatter tail of objects. In the current implementation, the parameters (*α*, *β*) are varied with the object size to realize the object-dependent scatter kernel. Initially, for both a cylindrical phantom (15 cm in diameter) and an IEC body phantom filled with uniform activity, we optimized the parameters (*α*, *β*) by matching the calculated scatter distribution with the tail part of the measured sinograms. Subsequently, the parameters were linearly interpolated and tabulated as a function of the object size. The volume of the object in the FOV is calculated from the segmented-transmission image (*μ* > 0.06 cm^−1^), and is used as the index of object size.

### Image analysis

All the reconstructed images (^15^O-H_2_O, ^15^O-O_2_, and ^15^O-CO) and parametric maps (CBF, CBV, OEF, and CMRO_2_) were spatially normalized to the anatomic brain template using the SPM tool (SPM8, http://www.fil.ion.ucl.ac.uk/spm, Wellcome Trust Centre for Neuroimaging, UCL, London, UK). Thus, the resultant images and maps were in the same data format with an isotropic voxel size of 2 mm.

Whole-brain analysis was performed on the reconstructed images to assess the overall trend of differences between the HDE and DEC methods. Separate masks were created for gray and white-matter (GM and WM) regions by thresholding (at > 0.5) the a priori tissue probability maps available in SPM. The GM and WM masks were applied to the reconstructed images from each patient. Mean values for the masked regions and differences in the mean values between DEC and HDE, (DEC − HDE)/HDE × 100 %, were calculated as a function of slice position.

For the parametric maps, the region of interest (ROI) analysis was performed: ROIs were defined on the template space, drawn bilaterally for each brain region (except for pons and midbrain) in 3 adjacent slices and results were averaged. Elliptical ROIs (16 × 32 mm) were defined for the cerebellar cortex, centrum semiovale, and 4 neocortical regions (frontal, temporal, occipital, and parietal). Circular ROIs (16 mm in diameter) were defined for the pons, midbrain, thalamus, putamen, parahippocampal gyrus, and cingulate gyrus (anterior and posterior parts). Clinical studies frequently use relative rather than absolute parameter values (e.g., relative to values in a normal brain region). We also analyzed the left-to-right ratios of the bilateral ROIs.

Intraclass correlation coefficient (ICC) was calculated with a two-way random effects model, ICC(2,1) [21], to evaluate the agreement between DEC and HDE.

## Results

**Fig. 2:**
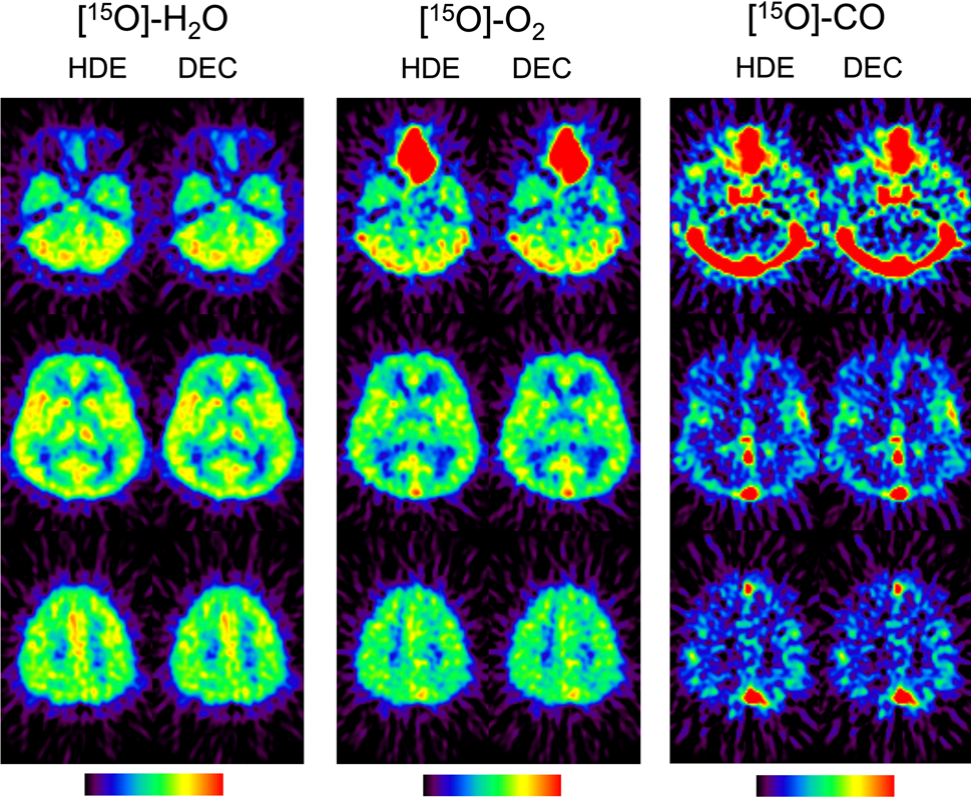
Representative reconstruction images, scatter-corrected by HDE and DEC, for ^15^O-H_2_O (*left*), ^15^O-O_2_ (*center*), and ^15^O-CO (*right*) from a patient with the stenosis of the left internal carotid artery (No. 10) and with three typical slice positions (subject’s native space). *HDE* hybrid dual-energy-window scatter correction, *DEC* deconvolution scatter correction

**Fig. 3:**
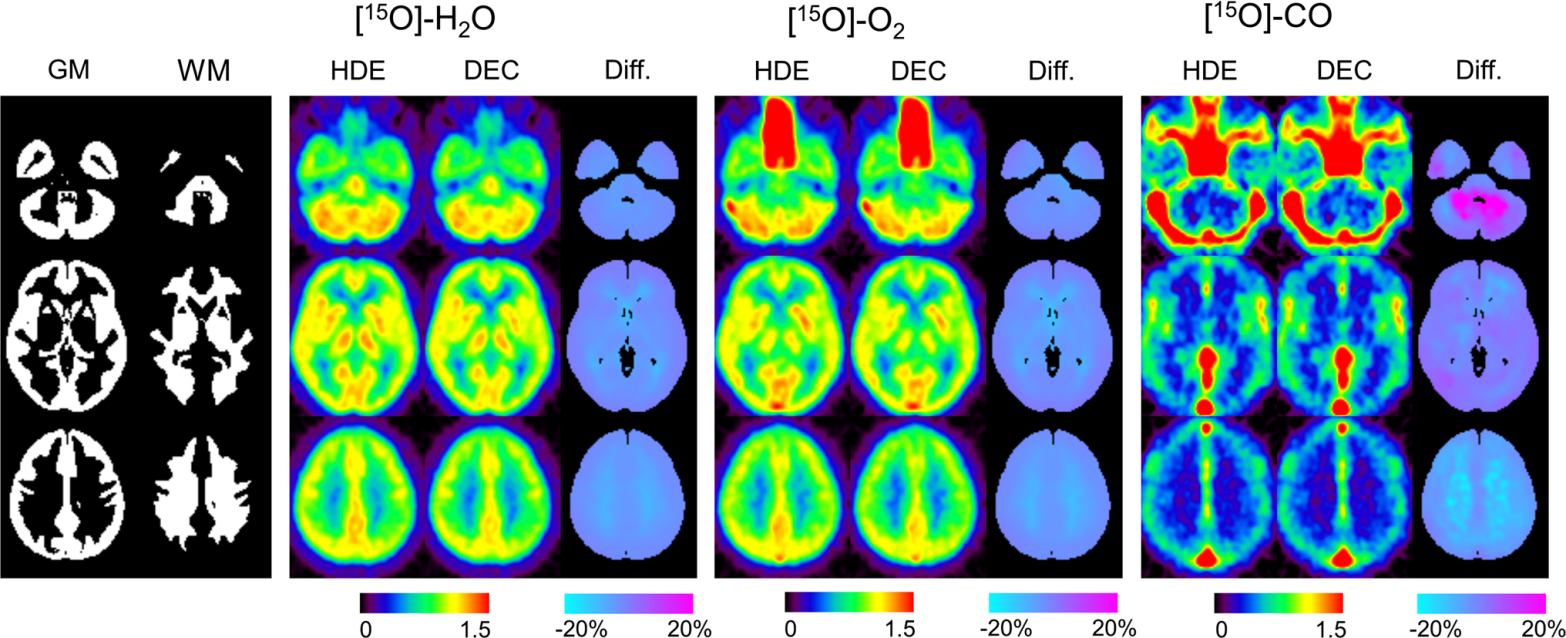
Patient-averaged reconstruction images, scatter-corrected by HDE and DEC, for ^15^O-H_2_O (*left*), ^15^O-O_2_ (*center*), and ^15^O-CO (*right*), with three typical slice positions. The spatially normalized images for each patient were normalized by the counts in GM regions and averaged over the patients. This count normalization was performed to visualize differences in GM-to-WM contrast between DEC and HDE. GM and WM masks, utilized for the analysis (Fig. 4), are also shown. *HDE* hybrid dual-energy-window scatter correction, *DEC* deconvolution scatter correction, *GM* gray matter, *WM* white matter

**Fig. 4:**
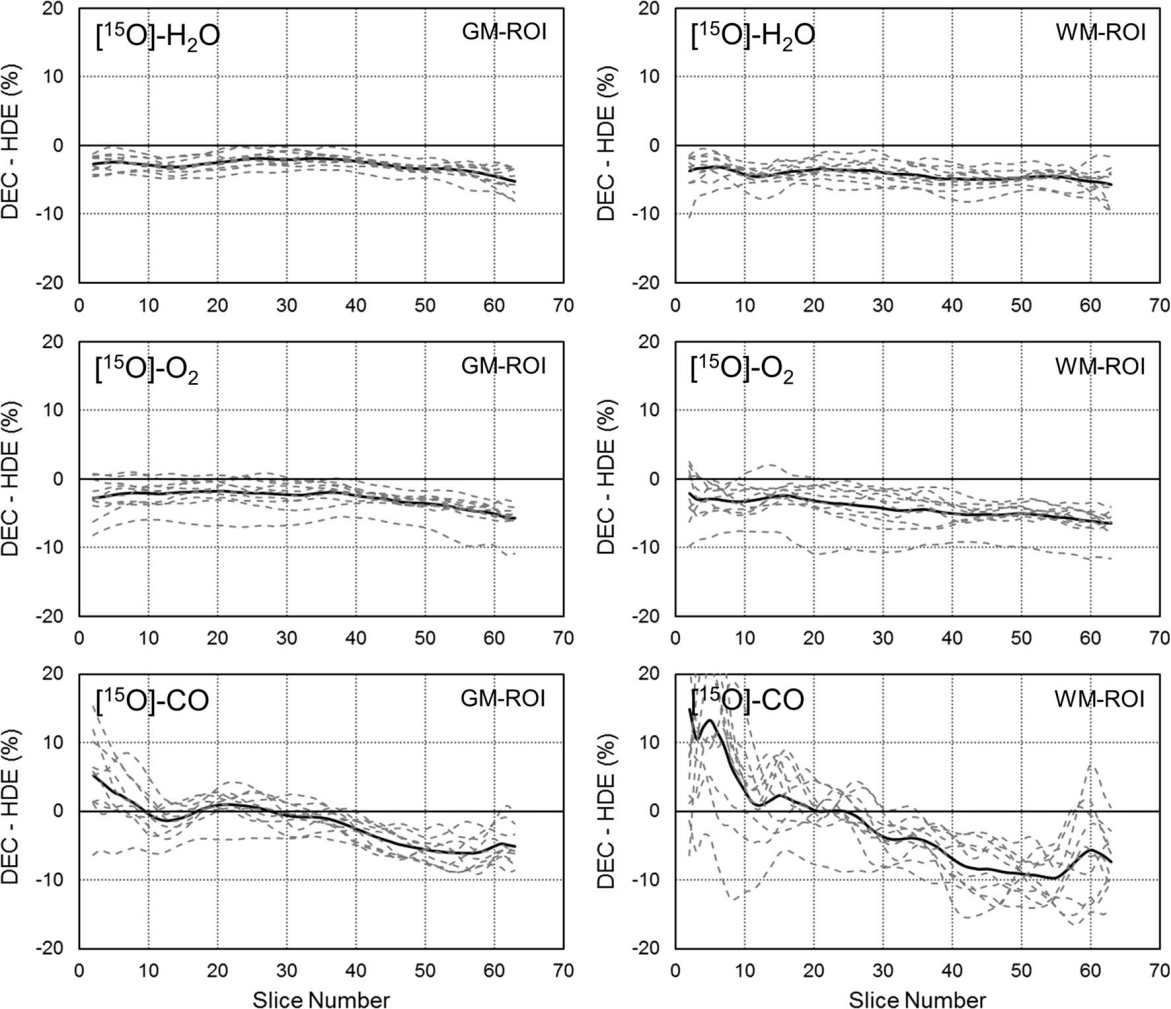
Differences in reconstruction images between DEC and HDE in % unit, defined as (DEC − HDE)/HDE × 100 %, are presented as a function of slice position: ^15^O-H_2_O (*upper*), ^15^O-O_2_ (*middle*), and ^15^O-CO (*lower*). The data were obtained from GM (*left*) and WM (*right*) regions, as shown in Fig. 3. *Solid lines* and *dotted lines* indicate the results for the patient-average and each patient, respectively. *HDE* hybrid dual-energy-window scatter correction, *DEC* deconvolution scatter correction, *GM* gray matter, *WM* white matter

Representative reconstruction images from a patient, scatter-corrected by HDE and DEC are shown for ^15^O-H_2_O, ^15^O-O_2_, and ^15^O-CO in Fig. 2. Visual assessment indicates that both the scatter correction methods provided similar reconstruction images with near-identical image noise. For detailed delineation of the differences between the methods, the reconstruction images for each patient were normalized by the counts for the whole GM region and then averaged over all the patients. The patient-averaged images, scatter-corrected by HDE and DEC, are shown in Fig. 3. The differences in the WM regions were slightly greater than in the GM regions, and were also greater for ^15^O-CO than for ^15^O-H_2_O and ^15^O-O_2_. However, as presented in Fig. 4 as a function of slice position, the differences for ^15^O-H_2_O and ^15^O-O_2_ were approximately 5 % in average even in the WM regions. The differences between the HDE and DEC methods were more substantial for ^15^O-CO, and showed greater slice-dependence, but values were generally still within 10 %, even in the lower slices.

**Fig. 5:**
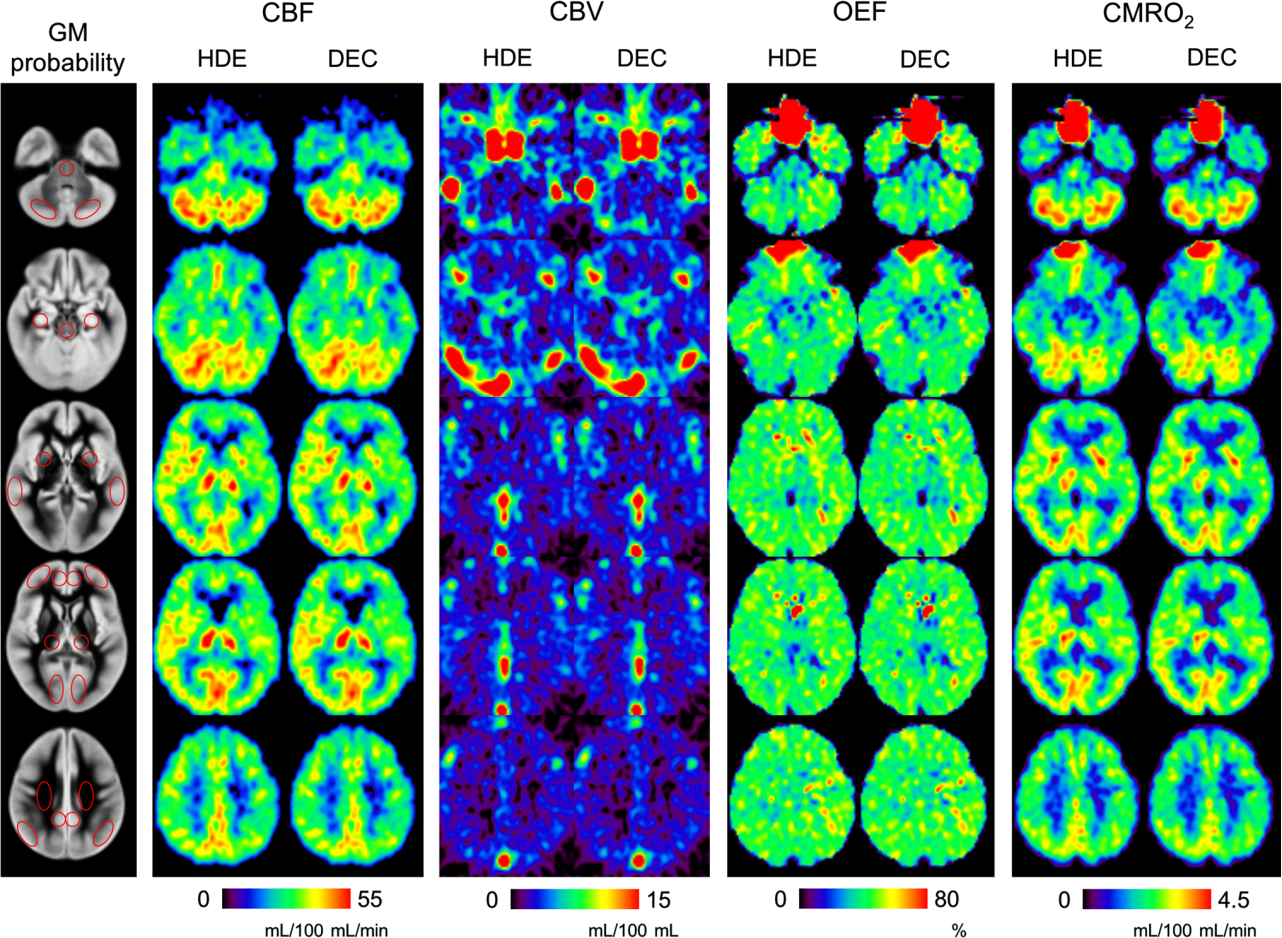
Representative parametric maps, calculated from the reconstruction images scatter-corrected by HDE and DEC, from a patient with occlusion of the left internal carotid artery (No. 3). Five typical slice positions are presented. Regions of interest overlaid on GM probability maps are also shown (*left*). *HDE* hybrid dual-energy-window scatter correction, *DEC* deconvolution scatter correction, *GM* gray matter

**Fig. 6:**
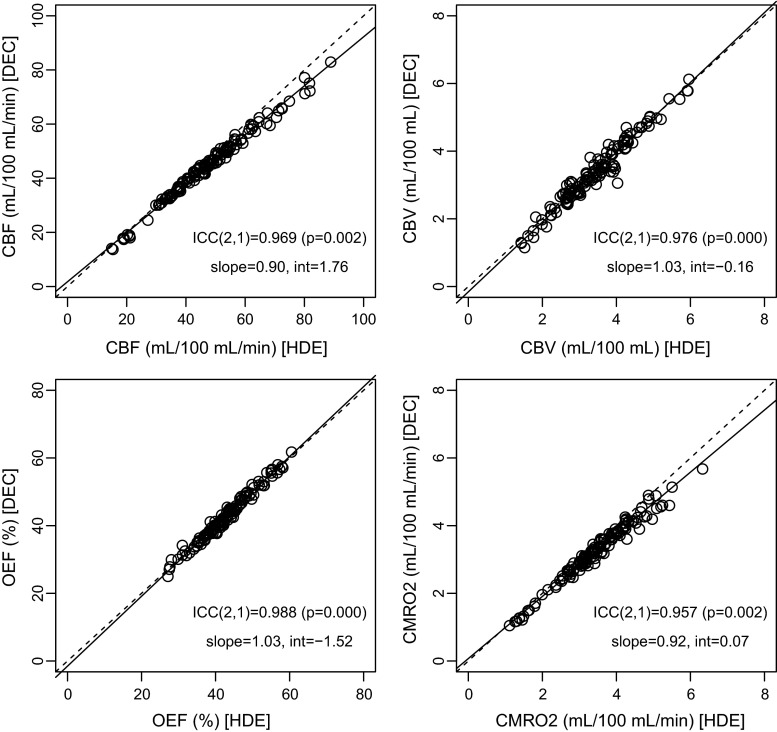
Correlations between HDE and DEC for CBF, CBV, OEF, and CMRO_2_. Each dataset has 130 data points (10 subjects × 13 ROIs). *Regression line* determined by geometric mean regression analysis (*solid line*), and line of identity (*dashed line*) are also shown. *HDE* hybrid dual-energy-window scatter correction, *DEC* deconvolution scatter correction, *CBF* cerebral blood flow, *CBV* cerebral blood volume, *OEF* oxygen extraction fraction, *CMRO*_*2*_ cerebral metabolic rate of oxygen

**Fig. 7:**
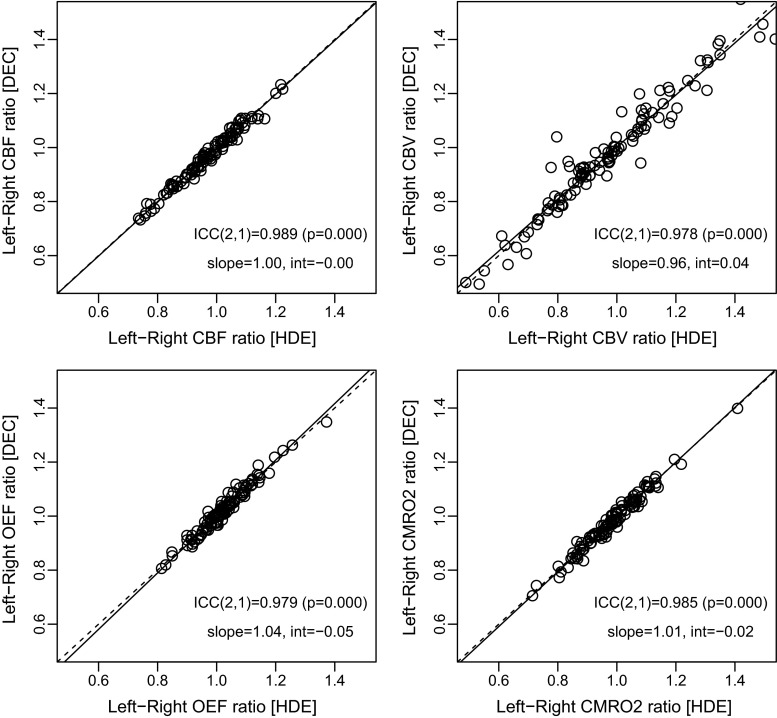
Correlations of left-to-right ratios between HDE and DEC for CBF, CBV, OEF, and CMRO_2_. Each data set has 110 data points (10 subjects × 11 ROIs). *Regression line* determined by geometric mean regression analysis (*solid line*), and line of identity (*dashed line*) is also shown. *HDE* hybrid dual-energy-window scatter correction, *DEC* deconvolution scatter correction, *CBF* cerebral blood flow, *CBV* cerebral blood volume, *OEF* oxygen extraction fraction, *CMRO*_*2*_ cerebral metabolic rate of oxygen

Representative parametric maps calculated from the scatter-corrected reconstruction images from a patient are shown in Fig. 5. Both scatter correction methods provided parametric maps of a similar quality, as was the case with the reconstruction images (Fig. 2). For the patient, whose images are shown (left ICA occlusion), a left hemisphere reduction in CBF and CMRO_2_, and slight increase in OEF, was visualized similarly by both HDE and DEC. The results of the ROI analysis comparing HDE and DEC are presented in Fig. 6. In addition to the absolute values, the relative values, as left-to-right ratios of each parameter, are also presented in Fig. 7. High ICC values were obtained for both the absolute and relative parameters, with an ICC > 0.95 for all the parameters (Figs. 6, 7). Absolute differences between DEC and HDE in % unit, defined as |DEC − HDE|/HDE × 100 %, are summarized in Table 1. The differences were not more than 10 %, except for quantitative CBV in the centrum semiovale (a WM region).

**Table 1:** Absolute differences between scatter correction methods: deconvolution (DEC) and hybrid dual-energy window (HDE)

Region	Absolute differences (%) in quantitative values				Absolute differences (%) in left-to-right ratios			
	CBF	CBV	OEF	CMRO2	CBF	CBV	OEF	CMRO2
Pons	6.3	7.3	3.5	7.6	–	–	–	–
Cerebellum	4.5	7.0	1.2	4.6	1.5	8.3	1.1	2.1
Parahippocampal gyrus	5.6	2.3	2.3	5.3	1.7	3.5	1.6	1.6
Midbrain	7.3	7.1	2.9	6.8	–	–	–	–
Putamen	6.8	4.6	2.0	8.1	1.7	5.3	1.1	1.3
Temporal cortex	2.6	2.7	1.5	3.4	0.7	1.6	1.6	1.3
Frontal cortex	2.0	2.2	1.6	2.8	1.2	2.8	0.8	1.1
Anterior cingulate	3.5	2.9	2.1	4.3	0.7	1.7	1.1	1.0
Thalamus	8.0	6.3	2.8	9.5	1.8	4.9	2.4	2.1
Occipital cortex	6.5	2.5	1.4	6.6	1.0	1.6	1.1	1.2
Posterior cingulate	7.1	5.7	1.4	7.4	0.6	1.5	0.9	0.8
Parietal cortex	3.8	4.1	1.3	3.8	1.2	3.8	1.2	1.8
Centrum semiovale	8.7	10.7	2.1	9.4	1.5	5.8	2.4	1.7
Total	5.6	5.9	2.0	6.1	1.2	3.7	1.4	1.5

Absolute differences, |DEC − HDE|/HDE × 100 %, are presented as patient-averaged values

## Discussion

The present study using ^15^O PET showed comparable results for the simplified DEC scatter correction method and the reference HDE method. The ^15^O PET study included three different scans, ^15^O-CO, ^15^O-O_2_, and ^15^O-H_2_O, each with a different distribution of radioactivity, and we consider it a good benchmark for evaluating the scatter correction method. The differences between the DEC and HDE reconstructed images were sufficiently small, generally in the range of 5–10 %, as presented in Fig. 4. Correspondingly, the ROI analyses also showed similar results for both the absolute parameters and the left-to-right ratios, thereby demonstrating the validity of the DEC scatter correction.

An intrinsic disadvantage of the calculation-based methods (e.g., DEC and SSS) is an inability to correct for scatter from outside of the FOV, in contrast to the energy-window-based methods (e.g., HDE). Nevertheless, the present ROI analysis showed similar results for both HDE and DEC, indicating that scatter from outside of the FOV is not a crucial problem for a clinical ^15^O brain study (at least under the settings used in this study), a finding that is consistent with previous studies [12, 13]. We have previously demonstrated that the use of the neck-shield reduces random coincidences originating from activity outside of the FOV. This results in significant improvement to the signal-to-noise ratio of the reconstructed images as confirmed by a bootstrap analysis [13, 22]. The study also showed that the reduction in scatter coincidences was small (around 5 %) [13], indicating that most scatter events come from activity inside the FOV, which are correctable by the calculation-based methods. On the basis of these findings, we conclude that calculation-based scatter correction methods, such as DEC, are practical for a clinical ^15^O brain PET scan with a 3D scanner. However, the SSS method [9, 10, 23], which is implemented in most commercial PET scanners, should be used with caution. Hori et al. [12] demonstrated that the currently implemented version of SSS [23] was inappropriate because of inadequate scaling to account for scatter from outside of the FOV, especially when used for ^15^O-gas PET. Rather, the original version of SSS, without a compensation procedure for scatter outside of the FOV, provided adequate results in the ^15^O-gas study [12].

The simplified implementation of DEC, i.e., the spatially-invariant, empirically-defined scatter kernel with optimized parameters for uniform phantoms, has an obvious limitation. Although uncertainty of HDE correction accuracy cannot be ruled out, the simplification and assumptions in DEC cause errors to various degrees. The scatter kernel naturally varies both with position in the scanner FOV and the objects being scanned [4, 7, 24, 25]. In the present study, the maximum differences between DEC and HDE were observed in a white-matter region at the level of the cerebellum in the ^15^O-CO images. These regions show a lower uptake of radioactivity and are sensitive to errors in the scatter correction because of a greater amount of scatter from surrounding regions with higher activity, such as large vessels. The situation may be more severe for lower parts of the brain with complex structures, on which image-based extraction of an AIF from a large artery (e.g., ICA) is a separate research topic [26–28]. For such regions, the simplified scatter kernel is a potential source of error. Unfortunately, the scanner used had a 156 mm axial FOV and had insufficient sensitivity for detailed investigations of the lower brain parts. A future study using a scanner with a longer axial FOV is desirable.

In the current implementation of DEC, object-size-dependent kernel parameters were applied to deal with objects of various size, and the DEC scatter correction is expected to work well for various applications, including whole-body scanning. In the present study with the ^15^O-labeled tracers, the phantom-based DEC parameters provided the satisfactory human results. However, there is a possibility of insufficient accuracy of DEC scatter correction for other PET tracers with radioactivity distribution extremely different to the ^15^O tracers, and further optimization of DEC parameters will be needed. In such a situation, optimization directly using human PET data instead of the uniform phantom data may be suitable although another additional human PET data are required for validation.

## Conclusions

The DEC scatter correction method despite its simplified implementation provides similar results to HDE when the fraction of scatter from outside of the FOV is sufficiently small. We consider it suitable for application in clinical ^15^O brain studies using 3D PET.

## References

[1] Frackowiak RS, Lenzi GL, Jones T, Heather JD (1980). Quantitative measurement of regional cerebral blood flow and oxygen metabolism in man using 15O and positron emission tomography: theory, procedure, and normal values. J Comput Assist Tomogr.

[2] Mintun MA, Raichle ME, Martin WR, Herscovitch P (1984). Brain oxygen utilization measured with O-15 radiotracers and positron emission tomography. J Nucl Med.

[3] Raichle ME, Martin WR, Herscovitch P, Mintun MA, Markham J (1983). Brain blood flow measured with intravenous H2(15)O. II. Implementation and validation. J Nucl Med.

[4] Bailey DL, Meikle SR (1994). A convolution-subtraction scatter correction method for 3D PET. Phys Med Biol.

[5] Grootoonk S, Spinks TJ, Sashin D, Spyrou NM, Jones T (1996). Correction for scatter in 3D brain PET using a dual energy window method. Phys Med Biol.

[6] Ferreira NC, Trebossen R, Lartizien C, Brulon V, Merceron P, Bendriem B (2002). A hybrid scatter correction for 3D PET based on an estimation of the distribution of unscattered coincidences: implementation on the ECAT EXACT HR+. Phys Med Biol.

[7] Bergstrom M, Eriksson L, Bohm C, Blomqvist G, Litton J (1983). Correction for scattered radiation in a ring detector positron camera by integral transformation of the projections. J Comput Assist Tomogr.

[8] Ollinger JM (1996). Model-based scatter correction for fully 3D PET. Phys Med Biol.

[9] Watson CC (2000). New, faster, image-based scatter correction for 3D PET. IEEE Trans Nucl Sci..

[10] Watson CC, Newport D, Casey ME, DeKemp R, Beanlands RS, Schmand M (1997). Evaluation of simulation-based scatter correction for 3-D PET cardiac imaging. IEEE Transac Nucl Sci..

[11] Ibaraki M, Miura S, Shimosegawa E, Sugawara S, Mizuta T, Ishikawa A (2008). Quantification of cerebral blood flow and oxygen metabolism with 3-dimensional PET and 15O: validation by comparison with 2-dimensional PET. J Nucl Med.

[12] Hori Y, Hirano Y, Koshino K, Moriguchi T, Iguchi S, Yamamoto A (2014). Validity of using a 3-dimensional PET scanner during inhalation of (15)O-labeled oxygen for quantitative assessment of regional metabolic rate of oxygen in man. Phys Med Biol.

[13] Ibaraki M, Sugawara S, Nakamura K, Kinoshita F, Kinoshita T (2011). The effect of activity outside the field-of-view on image signal-to-noise ratio for 3D PET with (15)O. Phys Med Biol.

[14] Matsumoto K, Kitamura K, Mizuta T, Tanaka K, Yamamoto S, Sakamoto S (2006). Performance characteristics of a new 3-dimensional continuous-emission and spiral-transmission high-sensitivity and high-resolution PET camera evaluated with the NEMA NU 2-2001 standard. J Nucl Med.

[15] Ibaraki M, Shinohara Y, Nakamura K, Miura S, Kinoshita F, Kinoshita T (2010). Interindividual variations of cerebral blood flow, oxygen delivery, and metabolism in relation to hemoglobin concentration measured by positron emission tomography in humans. J Cereb Blood Flow Metab.

[16] Hatazawa J, Fujita H, Kanno I, Satoh T, Iida H, Miura S (1995). Regional cerebral blood flow, blood volume, oxygen extraction fraction, and oxygen utilization rate in normal volunteers measured by the autoradiographic technique and the single breath inhalation method. Ann Nucl Med.

[17] Matsubara K, Ibaraki M, Nakamura K, Yamaguchi H, Umetsu A, Kinoshita F (2013). Impact of subject head motion on quantitative brain (15)O PET and its correction by image-based registration algorithm. Ann Nucl Med.

[18] Kanno I, Iida H, Miura S, Murakami M, Takahashi K, Sasaki H (1987). A system for cerebral blood flow measurement using an H215O autoradiographic method and positron emission tomography. J Cereb Blood Flow Metab.

[19] Iida H, Higano S, Tomura N, Shishido F, Kanno I, Miura S (1988). Evaluation of regional differences of tracer appearance time in cerebral tissues using [15O] water and dynamic positron emission tomography. J Cereb Blood Flow Metab.

[20] Martin WR, Powers WJ, Raichle ME (1987). Cerebral blood volume measured with inhaled C15O and positron emission tomography. J Cereb Blood Flow Metab.

[21] Shrout PE, Fleiss JL (1979). Intraclass correlations: uses in assessing rater reliability. Psychol Bull.

[22] Ibaraki M, Matsubara K, Nakamura K, Yamaguchi H, Kinoshita T (2014). Bootstrap methods for estimating PET image noise: experimental validation and an application to evaluation of image reconstruction algorithms. Ann Nucl Med.

[23] Watson CC, Casey M, Michel C, Bendriem B. Advances in scatter correction for 3D PET/CT. Nuclear Science Symposium Conference Record, 2004 IEEE: IEEE; 2004. p. 3008–12.

[24] Bentourkia M, Msaki P, Cadorette J, Lecomte R (1995). Assessment of scatter components in high-resolution PET: correction by nonstationary convolution subtraction. J Nucl Med.

[25] Lubberink M, Kosugi T, Schneider H, Ohba H, Bergstrom M (2004). Non-stationary convolution subtraction scatter correction with a dual-exponential scatter kernel for the Hamamatsu SHR-7700 animal PET scanner. Phys Med Biol.

[26] Su Y, Arbelaez AM, Benzinger TL, Snyder AZ, Vlassenko AG, Mintun MA (2013). Noninvasive estimation of the arterial input function in positron emission tomography imaging of cerebral blood flow. J Cereb Blood Flow Metab.

[27] Fung EK, Carson RE (2013). Cerebral blood flow with [15O]water PET studies using an image-derived input function and MR-defined carotid centerlines. Phys Med Biol.

[28] Iguchi S, Hori Y, Moriguchi T, Morita N, Yamamoto A, Koshino K (2013). Verification of a semi-automated MRI-guided technique for non-invasive determination of the arterial input function in ^15^O-labeled gaseous PET. Nucl Instrum Methods Phys Res A.

